# Engagnition: A multi-dimensional dataset for engagement recognition of children with autism spectrum disorder

**DOI:** 10.1038/s41597-024-03132-3

**Published:** 2024-03-15

**Authors:** Won Kim, Minwoo Seong, Kyung-Joong Kim, SeungJun Kim

**Affiliations:** Gwangju Institute of Science and Technology, School of Integrated Technology, Gwangju, 61005 South Korea

**Keywords:** Scientific data, Information technology, Electrical and electronic engineering

## Abstract

Engagement plays a key role in improving the cognitive and motor development of children with autism spectrum disorder (ASD). Sensing and recognizing their engagement is crucial before sustaining and improving the engagement. Engaging technologies involving interactive and multi-sensory stimuli have improved engagement and alleviated hyperactive and stereotyped behaviors. However, due to the scarcity of data on engagement recognition for children with ASD, limited access to and small pools of participants, and the prohibitive application requirements such as robots, high cost, and expertise, implementation in real world is challenging. However, serious games have the potential to overcome those drawbacks and are suitable for practical use in the field. This study proposes Engagnition, a dataset for engagement recognition of children with ASD (*N* = 57) using a serious game, “Defeat the Monster,” based on enhancing recognition and classification skills. The dataset consists of physiological and behavioral responses, annotated by experts. For technical validation, we report the distributions of engagement and intervention, and the signal-to-noise ratio of physiological signals.

## Background & Summary

Children with autism spectrum disorder (ASD) often exhibit delays in motor development and deficits in motor skills, which may compromise their engagement in daily activities^1,2^. These challenges have the potential to impede the development of cognitive, social interaction, and communication skills, exposing children with ASD to fewer learning opportunities^3,4^. Therefore, sensing and recognizing the engagement status of children with ASD is an important step that lays the groundwork for further interventions and promotes their improved engagement^5–9^.

Engaging technologies that involve physical activity^10–13^, interactive stimuli^14–16^, extended reality^12,17^, robot^18–21^ and serious games (SG)^22–24^ are beneficial to children with ASD by improving their engagement and reducing stereotyped behaviors. While physical activity has helped improve the engagement of children with ASD, recent technological advancements have allowed it to be applied to the fields of interactive digital treatment or SG incorporating motion tracking, and robotics. Many studies have focused on robot applications, such as robot-enhanced therapy and socially assistive robotics^18–21^, whose benefits include increased motivation and engagement, reduced anxiety compared to human interactions, and structured and repetitive supportâ€”particularly advantageous for engaging children with ASD^18^. Implementing robotic systems in the field, however, is challenging; it requires considerable expertise in robotics^25^ and high implementation costs^26,27^. These barriers reduce the feasibility of robot use for children with ASD in real-world settings.

Emphasis on artificial intelligence (AI) that is integrated with robots is growing, rather than solely relying on robot platforms. These AI techniques have the potential to solve challenging and complex problems in the field of robotics, particularly in real-world applications^28^ (e.g., detection of facial expression^29^ and emotional^18^ and stereotyped behavior^30^, and safety monitoring^31^). Applying an AI approach to SG that can be implemented in classroom settings is promising in terms of cost-effectiveness, lower barriers to implementation without expertise, and fostering problem-solving and learning skills, which can improve engagement and independence in children with ASD^22,32,33^. In addition, the primary advantages of robots (i.e., multi-sensory audiovisual aids and structured and repetitive support) can also be used in SG, and these can practically play to their strengths since applications with AI are part of their capabilities^18^. Thus, it is promising to promote SG incorporating AI to advance engagement recognition, “Engagnition” for further interventions.

Acquiring data and recruiting children with ASD, however, can be challenging owing to limited access and small pools of participants^6,34^. Consequently, few datasets focused on children with ASD exist and only a limited number of datasets are available for public use. While available datasets were published in the following areas: affective computing capable of recognizing emotions or stress (i.e., AKTIVES^35^, ReCANVo^36^), social interactions between conversational partners or robots^9,34^ (i.e., DREAM dataset^37^), and behavioral and gestural recognition^34,37^ for children with ASD, datasets relating to engagement relevant to learning opportunities and functional development are scarce as shown in Table 1. Research on the engagement of children with ASD is limited mostly by non-public datasets and focuses on robot applications, which results in high barriers to practical use. Additionally, these studies rely on limited-diversity datasets.

**Table 1:** Previous studies on engagement recognition in children with ASD and details of participants, interactions and tasks, dataset composition, engagement annotation (annotator), and lastly, data availability.

Study (year)	Participants (#)	Interaction and Tasks	Dataset Composition	Engagement annotation (annotator)	Data Availability
Jain *et al*.^29^	Children with ASD (*N* = 7)	Space-themed math game with socially assistive robot	Facial expression and detection confidence value, head position, eye gaze direction, audio feature, game performance and elapsed time	Engaged/ disengaged (the first author)	Public
Javed *et al*.^9^	Children with ASD (*N* = 5)	Sensory maze game with socially assistive robots	Video, audio and motion-tracking	0–5 Likert scale (3 human annotators)	On request
CultureNet^19^	Children with ASD (*N* = 30)	Robot-assisted autism therapy for emotion expressions	Face images from video	Continuous scale from −1 to +1 (5 experts)	Non-public
PPA-net^18^	Children with ASD (*N* = 35)	Robot-assisted autism therapy for emotion expressions	Facial expressions, head movements, body movements, pose, and gestures, audio, heart rate, electrodermal activity, body temperature	Continuous scale from −1 to +1 (5 experts)	Non-public
Chorianopoulou *et al*.^92^	Children with ASD (*N* = 9)	Conversational interaction between parents and child	Acoustic and linguistic data	Engaged/ disengaged (1 expert)	Non-public
Fan *et al*.^93^	Adolescents with ASD (*N* = 20)	Virtual driving	Electroencephalography, eye gaze	6-levels (1 expert)	Non-public
Engagnition* (Our dataset)^53^	Children with ASD (*N* = 57)	Serious game based on physical activity (low-and-high physical exertions)	Gaze fixation, intervention, GSR, ST, performance, elapsed time, ACC, SUS, NASA-TLX (Video and audio recordings)	3-levels (3 experts & the first author)	Public

Engagnition* in the last line represents the details of our dataset and study position within the field. Abbreviations are explained in the following paragraph.

Engagement is not a single attribute, but consists of multi-dimensional attributes in terms of the spectrum of engagement status: mental, behavioral, and emotional^6^. Thus, incorporating and integrating those that contain multi-dimensional sensors is crucial to ensure the Engagnition dataset. Our dataset includes expert annotations and physiological and behavioral responses: annotations on engagement^38,39^, gaze fixation^21,40^, and intervention^41^ and responses on galvanic skin response (GSR)^42–44^, skin temperature (ST)^45,46^, performance^47^, elapsed time^48^, and accelerometer (ACC)^49,50^. Additionally, we included subjective questionnaires to evaluate the system usability scale (SUS)^51^ and workload, called NASA Task Load Index (NASA-TLX)^52^.

**Fig. 1:**
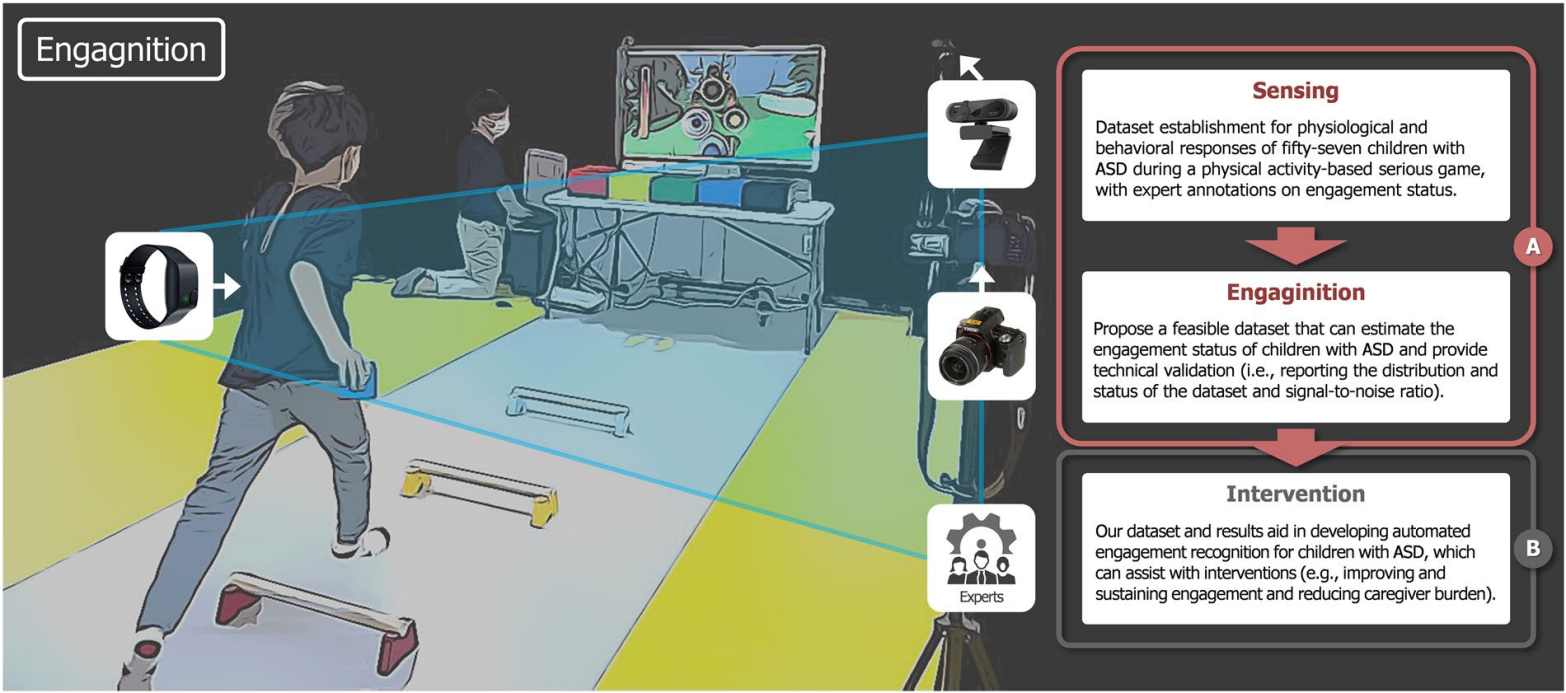
Overview of Engagnition dataset collection for children with ASD; Apparatus in the white squares, Empatica E4 wristband, and camera for expert annotation comprise Engagnition dataset; (**A**) the scope of dataset establishment covered in this paper (Sensing and Engagnition), (**B**) application areas where our dataset and results can contribute (Intervention).

Our study aims to compile the Engagnition dataset^53^ for children with ASD, during physical activity-based SG, and evaluate the distribution of engagement and intervention, and signal-to-noise ratio (SNR) on physiological signals for technical validations. As shown in Fig. 1, our Engagnition dataset^53^ contributes to the recognition of engagement for children with ASD during activities or SG with both static and dynamic physical activities using non-intrusive physiological and behavioral sensors with annotations by experts. Automating Engagnition with follow-up interventions may enable structured and repetitive learning from SG, improving the engagement of children with ASD and lessening the burden on special educators and parents^54^. In pedagogical applications for children with ASD, personalized and tailored intervention may enhance cognitive and motor development, leading to improved learning effects and skills. Lastly, gaining a better understanding of how children with ASD engage with Engagnition datasets^53^ could potentially lead to advances in engagement recognition and engage other researchers in this critical field of study.

## Methods

### Ethics statement

The study was approved and conducted according to the guidelines and regulations of the Institutional Review Board at Gwangju Institute of Science and Technology (approval number HR-61-04-04). The study to establish a dataset of physiological and behavioral responses, as well as subjective questionnaires for children with ASD, underwent a full review. Participants were informed about the study details, including the objectives, procedures, and the roles of parents and/or caregivers, and provided their consent to participate in the research. They agreed to be recorded during the study and consented to the use of personal data to the extent necessary for the research project.

### Participants

Fifty-seven children with ASD (age: *M* = 9.9; *SD* = 2.1, gender ratio: male = 40; female = 17) between 4 and 16 years old were recruited from “Dream Tree Children Education Center,” an institution that supports special education within the community and promotes special education on basic motor functions, physical strength, and mental and physical health. They ranged from low- to high-functioning ASD, and some participants were diagnosed with an intellectual disability (ID) (*N* = 23) and attention-deficit/hyperactivity disorder (ADHD) (*N* = 3). Details on demographics and individual properties of our study participants are illustrated in Table 2.

**Table 2:** Demographics and diagnostic properties of study participants across conditions for the Engagnition dataset^53^; LPE: low physical exertion, HPE: high physical exertion, P: participant, M: male, F: female.

Baseline				LPE				HPE			
P	Age	Gender	Diagnosis	P	Age	Gender	Diagnosis	P	Age	Gender	Diagnosis
P01	11	M	ASD	P20	11	M	ASD	P39	11	M	ASD
P02	9	M	ASD, ID	P21	9	M	ASD	P40	9	M	ASD, ID
P03	11	F	ASD	P22	10	M	ASD	P41	11	F	ASD
P04	10	F	ASD	P23	11	F	ASD	P42	10	F	ASD
P05	10	F	ASD, ID	P24	10	F	ASD	P43	10	F	ASD, ID
P06	10	F	ASD	P25	10	F	ASD, ID	P44	10	F	ASD
P07	4	M	ASD	P26	10	F	ASD	P45	10	M	ASD, ID
P08	13	M	ASD	P27	11	M	ASD, ID	P46	10	M	ASD
P09	10	M	ASD, ID	P28	6	F	ASD, ID	P47	8	M	ASD, ID
P10	10	M	ASD	P29	13	M	ASD	P48	13	M	ASD
P11	7	M	ADHD	P30	10	M	ASD, ID	P49	16	M	ASD
P12	11	M	ASD	P31	10	M	ASD	P50	12	M	ASD
P13	12	M	ASD, ID	P32	7	M	ADHD	P51	12	M	ASD, ID
P14	8	F	ASD, ID	P33	11	M	ASD	P52	10	M	ASD
P15	10	M	ASD	P34	12	M	ASD, ID	P53	10	M	ASD
P16	9	M	ASD, ID	P35	8	F	ASD, ID	P54	10	M	ASD, ID
P17	11	M	ASD, ID	P36	9	M	ASD, ID	P55	11	M	ASD, ID
P18	12	M	ASD	P37	12	M	ASD	P56	7	M	ADHD
P19	6	F	ASD, ID	P38	4	M	ASD	P57	8	F	ASD, ID

Parents of children with ASD volunteered to help in the dataset acquisition in response to the recruitment notice at the center. On the day of participation, children with ASD were accompanied by their parents or caregivers. Then they were separated into different spaces, with assigned roles: the study participant (i.e., a child with ASD) was in the data collection testbed, while the parent or caregiver was in the observation area for subjective assessment on the SG engagement of participant. This included the questionnaires of usability (i.e., SUS)^51^ and workload (i.e., NASA-TLX)^52^ assessment, which are completed by proxy users (i.e., their parent or caregiver) familiar with the study participants. Proxy users performed assessments on behalf of children with ASD for more reliable outcomes^55,56^. For the role in dataset acquisition, a compensation of $75 was provided to the parent or caregiver, including a reward for the child with ASD.

### Serious game, “Defeat the Monster.”

To formulate an SG tailored for children with ASD for the Engagnition dataset, we interviewed a set of experts as an initial design step and conducted a series of iterative pilot studies^57^. The initial design process involved selecting the theme of the SG, designing the tasks to be performed in the game, and determining different levels of difficulty. The feedback and insights derived from expert interviews were then incorporated into a theoretical approach to develop an engaging SG^23,58,59^. The series of pilot studies was designed to verify the usability and applicability of the SG. The group of experts consulted in this initial step comprised 10 teachers with over 13 years of collective experience in on-site special education with students with ages between from 4 and 16 years to match the age ranges represented in the dataset.

In developing the SG, we considered that suiting the target population and aligning well with their levels of skill were crucial concerns. The target population of our study group was defined as children with ASD aged 4 years and older, which falls within age ranges (≥4 years) for which such games are considered feasible. For example, exergames with more rigorous physical exertions such as dancing^60,61^ have been investigated, whereas the SG described here only includes walking and classifying activities. Similarly, an SG was also investigated that required more demanding interaction with a virtual character for social communication^32^ compared to the interaction demands of our SG (i.e., recognizing and classifying activities). Therefore, the SG implemented for the Engagnition dataset was designed for children with ASD aged between 4 and 16 years.

We developed “Defeat the Monster” as an SG designed to emulate the Nintendo Ring Fit. It involves two different levels of physical exertion, designated as low (LPE) and high physical exertion (HPE), which include both static and dynamic activities. Applying exergames (i.e., videogames that involve physical exertion^62^) in SGs has been a topic of research to support increased engagement and motivation in children with ASD^23,58,63–65^ given their applicability and simplicity in implementation. With evident benefits in reducing repetitive behaviors as well as improving motor skills and behavior on executive function tasks^64,65^, exergames have been adopted in tandem with learning activity in the classroom^66^. Their practical applications have expanded to in-class activity and pedagogical methodology (based on a consensus of more than eight experts) using commercial games such as Nintendo’s Ring Fit Adventure^62^. In this study, the SG was designed to enhance cognitive and motor development by incorporating visual perception, motor planning, and execution of movements through the recognition and classification of toy blocks^67,68^.

**Fig. 2:**
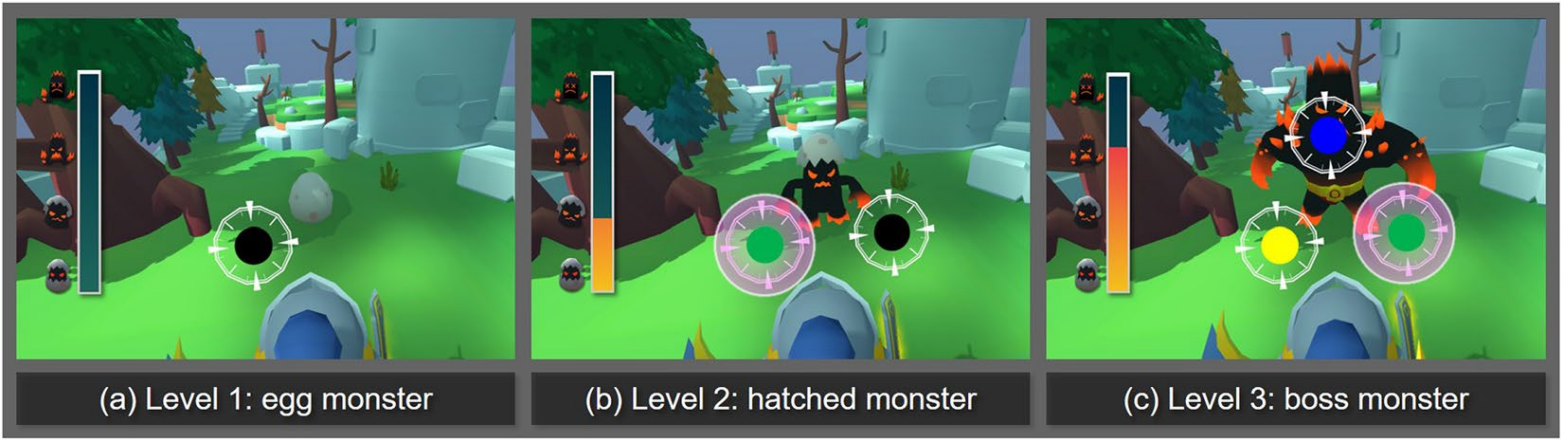
Presentation of SG in-game elements (i.e., monster and given tasks) according to the level of difficulty.

The SG begins with a storyline where players defeat a monster by throwing colored balls at it. To attack the monster presented on the screen, players must recognize the highlighted effect on the monster, then pick up the same color toy block from those randomly placed on a desk in front of the screen (i.e., LPE) and far away from the screen (i.e., HPE), and classify it into a box of matching color on the desk right in front of the screen. Five colors are provided: red, yellow, green, blue, and black, and a highlighted effect is randomly assigned to each trial^35,69^. Every time a color is presented on the screen, a highlighted effect appears on the weak point of the monster. Each time an attack is made by classifying the toy block, the score is calculated and the anger gauge of the monster increases. The level of difficulty varies based on the acquired score, as illustrated in Fig. 2 and Table 3. When the anger gauge reaches its maximum, the SG finally ends.

**Table 3:** Differential scoring logic and in-game elements applied to SG according to the level of difficulty.

Level of difficulty	Score range	Score (success/failure)	Monster type	Number of colors given by task
Level 1	0–20	+10/+5	Egg monster	1-color (Fig. 2(a))
Level 2	20–40	+4/+2	Hatched monster	2-colors (Fig. 2(b))
Level 3	40–60	+2/+1	Boss monster	3-colors (Fig. 2(c))

The level of difficulty was elevated by increasing the number of task colors presented on the screen that the players were assigned to recognize for classification in three levels^70,71^ and decreasing reward points^70^ as shown in Table 3. Increasing levels of difficulty is important in designing SGs because it leads to increasing competence in particular skills for children with ASD by providing challenging yet achievable goals in given tasks^58,72^. Thus, defining well-mediated levels of difficulty is crucial to prevent children with ASD from becoming frustrated by drastic changes such as situations where levels of difficulty change rapidly or the type of gameplay becomes inconsistent, as one of the experts pointed out. Involving more distractors in a series of distinct stages^59,73,74^ and increasing the speed of the game^75,76^ are prevalent techniques to increase levels of difficulty in SGs for children with ASD. As the goal of our SG was to enhance recognition and classification skills^67,68^, the level of difficulty was designed to increase gradually by presenting more colors at each level from 1 to 3 by adding more visual distractors and aggravating the perceptual demands on the player^59,71,77^. The difficulty increases in levels based on defined ranges of cumulative scores^58^. Stronger-looking types of monsters appear at higher levels of difficulty; for example, an egg monster appears on difficulty level 1, a hatched monster appears on level 2, and a boss monster appears on level 3. Reward scores also decrease progressively at higher difficulty levels^70^. The scoring logic is designed to grant points even if the player fails (i.e., no decrease in points for failed tasks)^78^ to prevent children from being frustrated and discouraged by failing to hit the monster’s weak point. Levels of difficulty were not counterbalanced due to the hypersensitivity of children with ASD^79–81^.

An iterative series of pilot studies was conducted to ensure the applicability of our SG for children with ASD before data were collected, initially with groups of typical participants and subsequently with experts and children with ASD. The first pilot study was conducted to identify and verify usability issues from the perspective of two typical participants (ages 20 and 23) with backgrounds in the field of human-computer interaction from undergraduate and graduate courses. A follow-up pilot study was conducted at an on-site location where data were collected. This involved three experts with over five years of experience in special education and two children with ASD at the median age of the Engagnition dataset (9 and 10) to discover any on-site difficulties and problematic issues that should be improved and verify that our SG was accessible and feasible for children with ASD.

The first pilot study identified some areas of improvement for the animation displayed when the player throws a toy block. The animation was complemented to provide visual feedback on accuracy by showing a trajectory for the animated toy^58,82^. This is, if an attack on the monster’s weak point is successful, the toy is shown hitting the monster on the screen with a sound effect indicating success. If an attack fails, the toy is shown passing to the side of the monster with a sound effect indicating a miss. Key findings from a follow-up pilot study indicated that the size of highlighted area showing the monster’s weak point and that of the interface elements showing different target colors in the original version might not be accessible and clear for children with ASD. The highlighted effect presented as the monsters’ weak point was shown in white in the initial version of the game. We changed this to purple as shown in (b) and (c) in Fig. 2 to avoid overlapping with our five task colors (e.g., red, yellow, green, blue, and black), because the white color could be difficult to see and discern for children with difficulty with visual sensory cues^83,84^. The size of the circle showing the color was therefore increased to improve accessibility and clarity to support the children in focusing on the targeted objectives^85^. Thus, we confirmed that young children with ASD (4 and 6 years old), including the youngest children, had no difficulties understanding and playing the SG in the main data collection process through iterative modifications in a series of pilot studies.

### Apparatus

#### Engagnition sensors and condition setups

**Fig. 3:**
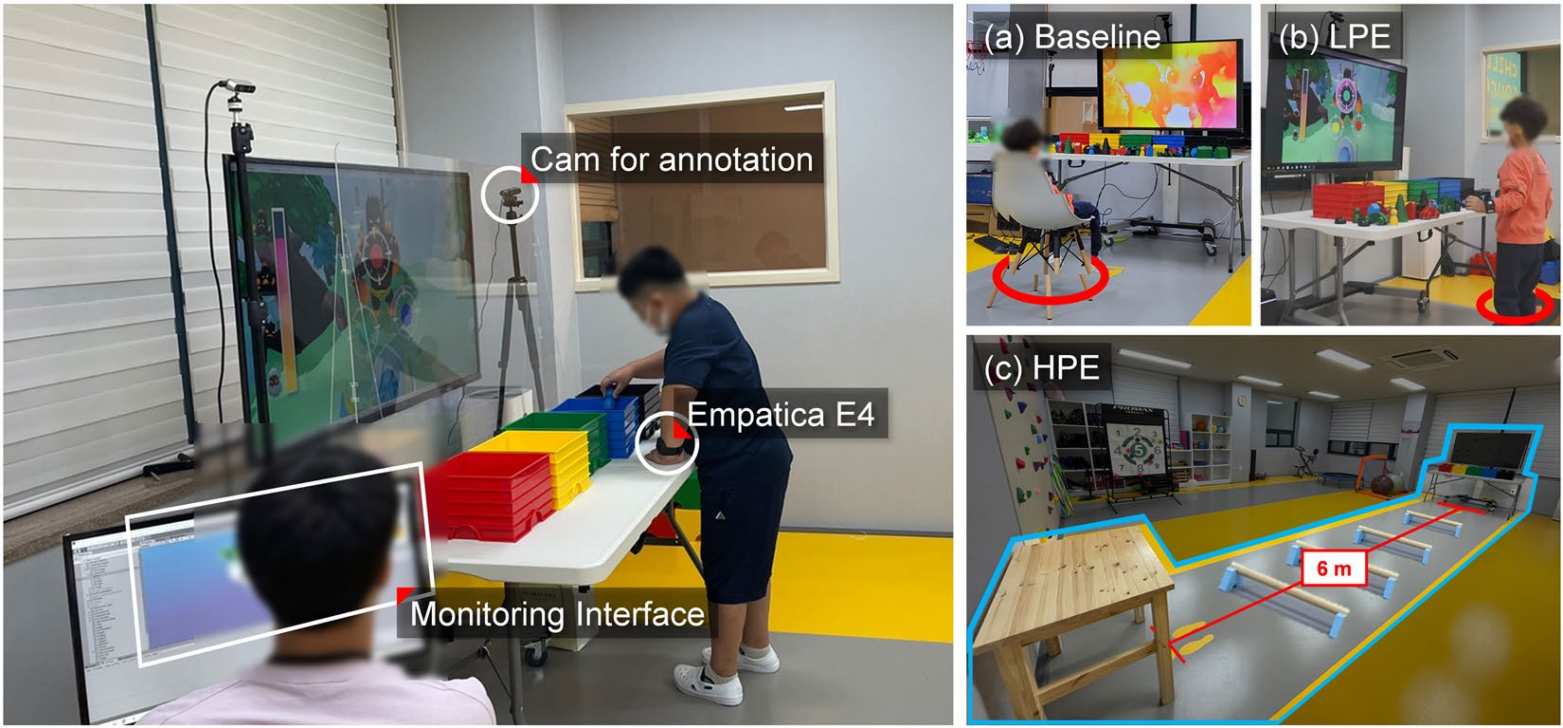
Experimental setup for dataset acquisition under the following conditions: (**a**) baseline, (**b**) LPE, and (**c**) HPE.

Owing to the hypersensitivity of children with ASD, non-intrusive wearable wristbands and web camera sensors were used as shown in Fig. 3. The Empatica E4 wristband was attached to collect time-series data (i.e., ACC, GSR, and ST)^86^. These data streams were presented in real-time to experimenters for supervision and control purposes, along with a game running scene on the right, as shown in Fig. 3. Front and rear cameras were set up to record and observe the behavior of the children during SG. These recordings were later used to annotate engagement, gaze fixation, and intervention purposes.

Experimental setups for data collection on three conditions, baseline, LPE, and HPE, were designed differently as shown in Fig. 3. The baseline data were collected for a minute, to achieve the state of maximum relaxation and minimize any effects from physical exertion after sensor stabilization. In the baseline, children sat in chairs and watched videos on YouTube based on their preferences or suggestions from parents and therapists^87^ as shown in Fig. 3(a). In LPE, participants stood in place and performed SG by recognizing the color presented on the screen and classifying toy blocks into the box of the same color, as shown in Fig. 3(b). HPE demands higher physical activity from the basis of LPE (i.e. the same SG). For instance, HPE involves walking back and forth over a 6-meter area with four obstacles positioned at 1.2-meter intervals as shown in Fig. 3(c). These obstacles were additionally included by therapist, who mentioned, “This would make it more familiar, increase the intensity of physical exertion, and make it more helpful in engaging the children by including instruments already in use.”

#### Engagnition annotation tool

**Fig. 4:**
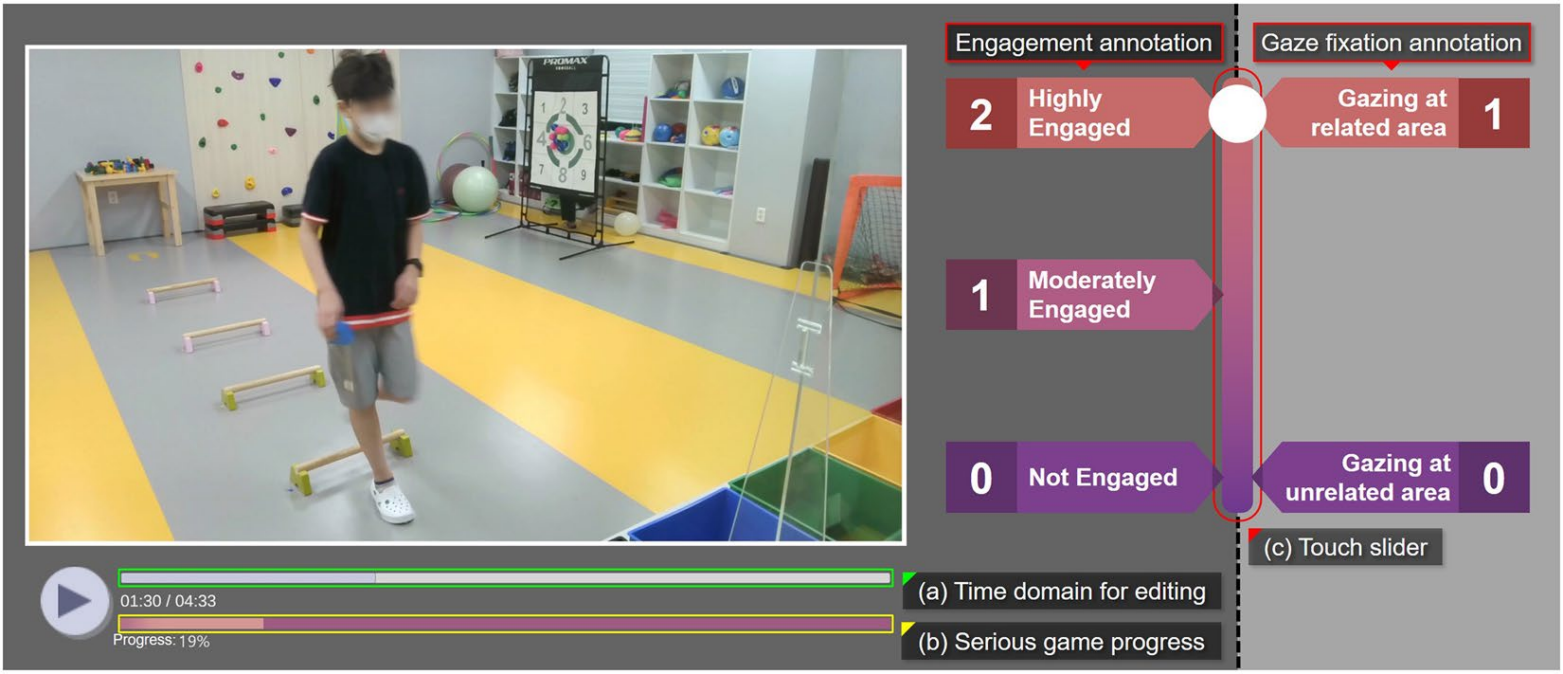
Our own-developed annotation tool for engagement and gaze fixation for children with ASD. Our tool features the following functions: (**a**) time domain for editing the annotation, (**b**) overall progress of SG, and (**c**) touch slider.

We developed our own annotation tool for engagement and gaze fixation as shown in Fig. 4. This tool allows annotators to annotate using a touch slider, enabling immediate and linear annotations. It responds quickly to behavioral changes and retains the touch input. In addition, an editing function that allows pausing or returning to a previous point was applied for adjustments.

**Fig. 5:**
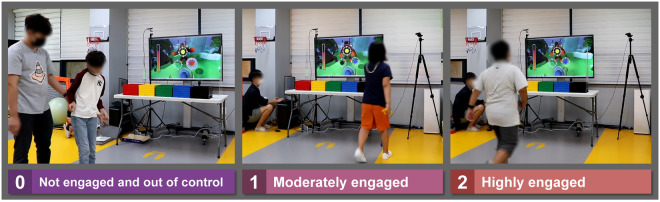
Snapshot of annotated engagement of children with ASD during the SG, from left: low-to-high engagement status.

Engagement was annotated using a ternary code to serve as the ground truth for how children with ASD engaged in a given SG. To ensure reliable engagement annotation, we grouped the annotators into a team of four, which consisted of three experts with over five years of experience, working as therapists for the participants, as well as a researcher for annotation mediation and supervision. Figure 5 depicts a snapshot of an annotation interface of an expert, illustrating how the engagement of children with ASD was annotated during SG.

Gaze fixation was annotated using a binary code to determine whether children with ASD fixated their gaze on the relevant areas of the SG. These are the essential areas that require focused gazes for navigating and progressing during SG, which involve a screen that presents SG, colored-toy blocks, and colored boxes. Before annotation, these criteria were predefined to ensure consistency in annotation. Initially, we considered measuring gaze fixation with a bar and glasses-type sensors; however, we substituted with annotation owing to the following reasons: the limited coverage of the entire action area (bar), hypersensitivity, and concerns about introducing new equipment and its potential impact on performance (glasses).

### Measures

**Fig. 6:**
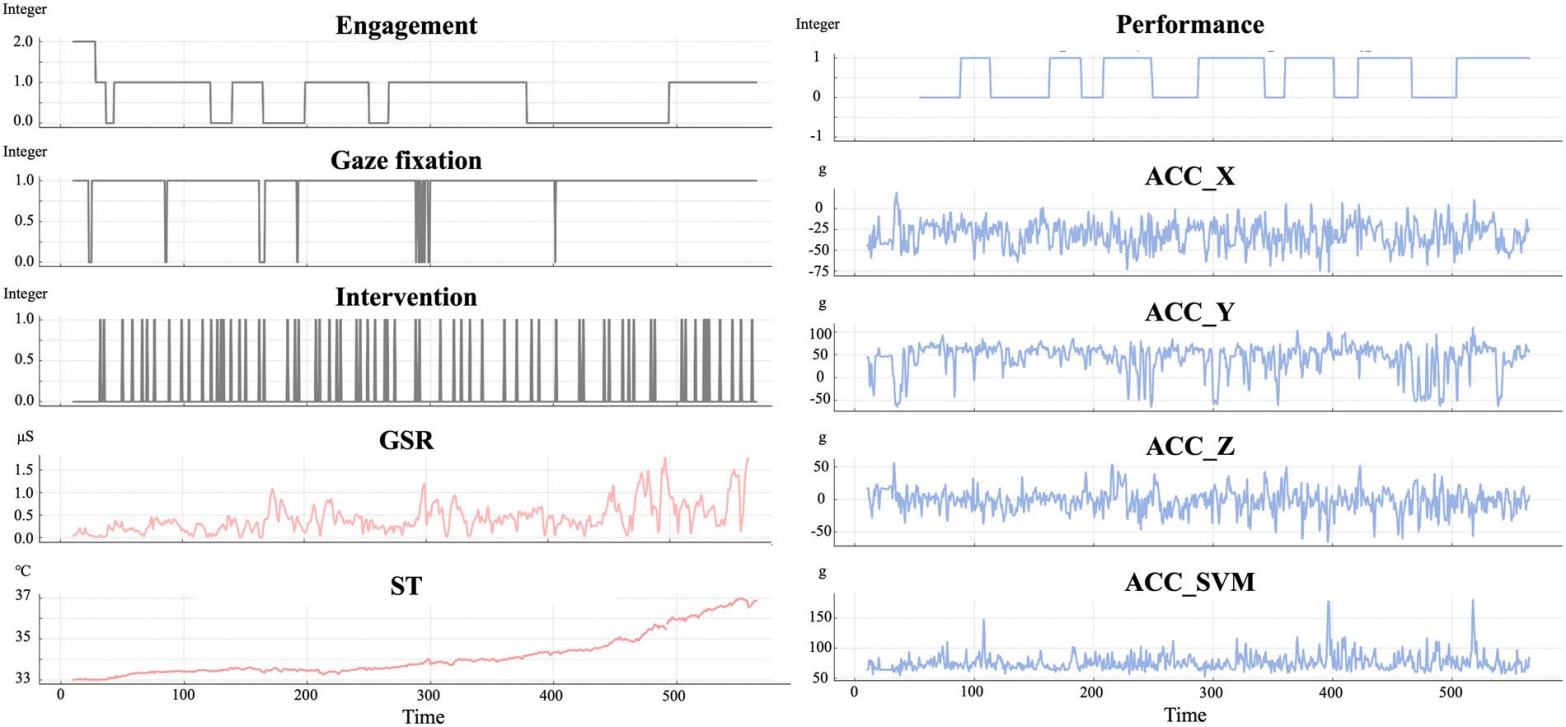
Data stream visualization of the Engagnition dataset^53^. These data are samples from one of the participants; the gray graph indicates annotation data, the red graph signifies physiological data, and the blue graph denotes behavioral data.

We established a dataset within four categories (e.g., annotation, physiological and behavioral response, and a subjective questionnaire) as shown in Table 4 and Fig. 6. Categorized data or signals are shown with the sampling rate, ranges from minimum to maximum (if possible), and the unit of each data type. Column names, which can be found in our dataset, are illustrated. These are provided in CSV format in time-series, except spent time and subjective questionnaire, which cannot be described in time-series.

**Table 4:** Detailed descriptions of the Engagnition dataset^53^ and the properties of each data or signal.

Data category	Data/signal	Sampling rate	Range	Unit	Column names in dataset
Annotation	Engagement	60 Hz	0/1/2	Integer	Engagement
	Gaze fixation	60 Hz	0/1	Integer	Gaze
	Intervention	*On each occasion*	—	Time stamp	Time stamp and type
Physiological response	GSR	4 Hz	—	*μ*S (Siemens)	GSR
	ST	4 Hz	—		Tmp
Behavioral response	Performance	*Every session*	0/1	Score	Performance
	Elapsed time	*Every session*	—	s (second)	Elapsed time in each session
	ACC	32 Hz	—	g ≈9.81*m*/*s*^2^	X, Y, Z, SVM
Subjective questionnaire	SUS	*Post-experiment*	0–100	Score	Usability score
	NASA-TLX	*Post-experiment*	0–100	Score	Workload score

Our annotations consist of three components: engagement, gaze fixation, and intervention. First, engagement, serving as a ground truth for how participants engaged in the SG was annotated with three-fold divisions^38,39^: 0 — not engaged and out of control, 1 — moderately engaged, 2 — highly engaged. Gaze fixation was annotated based on the gaze of participants that stayed on the SG-relevant area: 0 — gazing at areas unrelated to SG, and 1 — gazing at related areas^88,89^. Exceptions were made when gazing to confirm or agree with the therapist and experimenter about performing the SG, which was also classified as 1. Lastly, the time stamps of intervention required^41^ for participants to proceed with SG were annotated with intervention types (e.g., discrete intervention with time stamps, continuous intervention for all time, and no need for intervention).

Our physiological responses included GSR^42–44^ and ST^45,46^. GSR, often referred to as skin conductance, gauges the electrical resistance of the skin, which changes based on its moisture levels. GSR serves as an indicator of sympathetic arousal, measuring levels of attention and affective states, which are also associated with engagement^42,43^ of participants during SG. ST provides an understanding of the surface thermal response of the body, which reacts according to changes in blood flow due to vascular resistance or arterial blood pressure^45,46^. ST is also associated with the arousal and attention state of the study participant. Therefore, ST was used to offer an additional layer of physiological data, complementing GSR.

Behavioral responses included performance^47^, elapsed time^48^, and ACC. Performance, as an indicator of accuracy, denotes whether the participant successfully completed the process of recognizing the color presented and classifying the toy block into the same-colored box during each session (i.e., success/failure). This data was automatically generated from log files, and the elapsed time for each session was also recorded. ACC based on the 3-axis (x, y, z) was collected to quantify active movement, which is related to higher ACC. To capture and generalize the intensity of these movements, signal vector magnitude features were extracted from the ACC data by calculating the square root of the sum of the squared components of the vectors^90,91^.

Lastly, the subjective questionnaire consisted of SUS^51^ to gauge the usability and NASA-TLX^52^ for workload assessment. A higher score on the SUS indicates better usability, while a higher score on the NASA-TLX indicates a higher workload.

### Procedures

Our Engagnition dataset^53^ was established with three sequences: main data collection, post-questionnaire, and annotation. Before the main data collection, the study participants were informed about the storyline of SG, and were given a tutorial on how to navigate and complete the SG. They had practice sessions to familiarize themselves with SG and the setup for E4 sensors to their wrist, to stabilize sensor signals before the main data collection. Finally, the dataset for physiological and behavioral responses was compiled with a randomly assigned order on conditions of baseline, LPE, and HPE.

After the questionnaire, datasets for the subjective questionnaire (SUS and NASA-TLX) were compiled by caregivers and parents on behalf of the participants at the end of data collection. During the main experiment, caregivers or parents observed the SG participation of the participant through a window and a tablet PC that provided real-time mirroring. Based on those observations, they were asked to evaluate subjective questionnaires with either format of the documents (printed material) or web survey platform (SurveyMonkey).

Finally, the dataset for annotations was obtained through post-video analysis after the experiment. To ensure reliable annotations, we involved experts who have over five years of experience in the field and work as therapists for our study participants. They were familiar with the study participants and were able to better understand their intrinsic characteristics and overall performance. The therapist in charge, who participated in the data collection, primarily manipulated the touch slider, and other annotators observed together to compile, discuss, and finally modify the engagement and gaze dataset. Where any team member objected, the annotations in the data stream that caused objection were re-examined until a consensus was reached among all team members. In the case of the interventions, time stamps of intervention were generated from the actual number of interventions provided during SG and potential additional interventions required by experts.

## Data Records

The Engagnition dataset^53^ can be accessed on figshare. We established the multi-dimensional Engagnition dataset^53^ spanning 270.6 minutes from 57 participants, each exhibiting varying symptoms of ASD. Data were gathered under baseline, LPE, and HPE conditions. The baseline condition consisted of 64.88 minutes, averaging 3.41 minutes per participant with a standard deviation of 0.53 minutes. The LPE data spanned 69.60 minutes, with an average contribution of 3.66 minutes per participant and a standard deviation of 1.80 minutes, whereas the cumulative duration of HPE data was 136.08 minutes, with participants contributing an average of 7.16 minutes, with a standard deviation of 2.62 minutes.

**Fig. 7:**
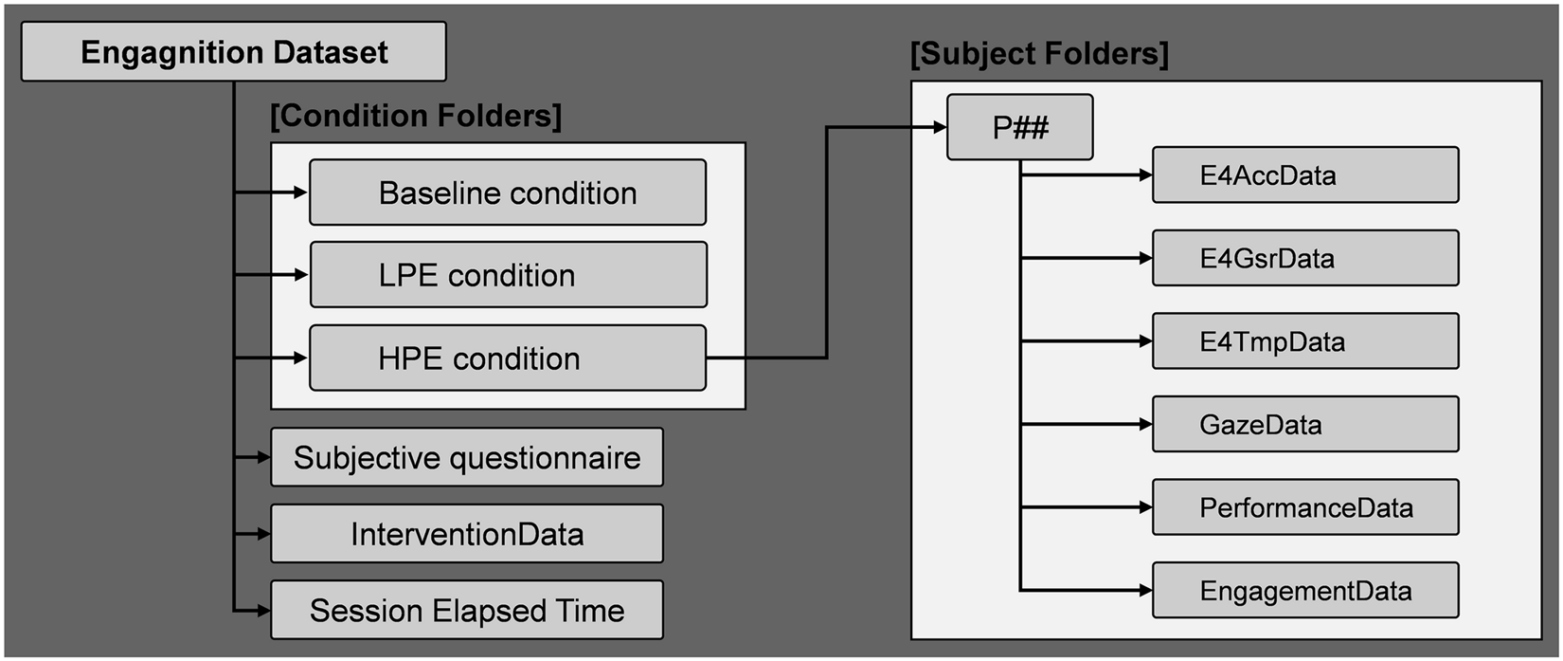
Visual representations on the structure and folder for Engagnition dataset^53^.

All the data are stored in both CSV and XLSX formats, with the data of each participant organized within individual folders. The organization of the dataset is illustrated in Fig. 7, which provides a visual representation of the structure of the dataset. The dataset is presented with hierarchical structure, starting with the top-tier directory labeled “Engagnition Dataset.” Within this primary folder, there are three general files: a subjective questionnaire file, an interventionData file, and a file noting the elapsed time in each session. Specific folders can be used under different conditions. Within these condition-specific folders, sub-folders designated by participants identification numbers contain individual datasets, such as E4AccData, E4GsrData, E4TmpData, GazeData, PerformanceData, and EngagementData. Notably, the Baseline does not have SG participation, thus it does not involve the annotation of engagement, gaze fixation, and intervention, the behavioral responses of performance and elapsed time, and the subjective questionnaires, SUS and NASA-TLX, due to the incomparable nature between SG and the interaction of the baseline established for the maximum relaxation condition. Our dataset is accessible in the dataset repository. A concise overview of the data types available is presented in Table 4. For more detailed specifications and descriptions of the data, please refer to the accompanying README.txt file.

### CSV file details

The CSV file comprises Unix timestamps for the dataset and corresponding data streams. The subsequent subsections delve into the specifics of each data stream.

#### E4AccData.csv

This dataset encompasses accelerometer values for the non-dominant hand, expressed in *m*/*s*^2^, across six rows. The first row denotes the SG timestamps, the second row is designated for the Unix timestamps, the third row is designated for the X values of the accelerometer, the fourth row corresponds to the Y values, the fifth row is allocated to the Z values, and the sixth row contains the signal vector magnitude values.

#### E4GsrData.csv

This dataset encompasses GSR values for the non-dominant hand, expressed in micro Siemens (*μ* S) units, across three rows. The first row denotes the SG timestamps, the second row contains the Unix timestamps and the third row contains the GSR values.

#### E4TmpData.csv

This dataset encompasses ST values for the non-dominant hand, expressed in degrees Celsius (), across three rows. The first row denotes the SG timestamps, the second row represents the Unix timestamps and the third row contains the ST values.

#### GazeData.csv

This dataset encompasses gaze fixation annotation values, expressed in 0 or 1 integers, across two rows. 0 denotes the absence of visual attention directed towards the SG-related area, whereas 1 signifies the presence of visual attention directed towards the SG-related area. The first row contains the SG timestamps, and the second row contains the gaze fixation annotation values.

#### PerformanceData.csv

This dataset encompasses performance annotation values, expressed in 0 or 1 integers, across two rows. 0 represents execution failure, while 1 signifies execution success in SG. The first row contains the SG timestamps, and the second row contains the performance values.

#### EngagementData.csv

The dataset contains engagement annotation values, which are represented by integers 0, 1, or 2, laid out across two rows. The first row contains the SG timestamps, and the second row contains the engagement values. The notation is such that 0 represents no engagement, 1 indicates moderate engagement, and 2 signifies high engagement.

### XLSX file details

The provided XLSX file incorporates three main categories: ‘Subjective questionnaire’, which captures usability and workload by the proxy user; ‘InterventionData,’ marking the time stamps when the intervention is required; and ‘Session Elapsed Time,’ offering time duration spent in each session. The following subsections will delve into the specifics of each data category.

#### Subjective Questionnaire.xlsx

The ‘Subjective questionnaire’ section captures responses from both the SUS and NASA-TLX, with distinct datasets organized into separate tabs for clarity. For the NASA-TLX, columns represent the experimental ‘Condition,’ ‘Participant’s identification number,’ scores for individual items, and both â€˜Unweighted’ and ‘Weighted’ aggregate scores. Similarly, the SUS dataset outlines the ‘Condition,’ ‘Participant’s identification number,’ individual query scores, and the final ‘Total Score.’

#### InterventionData.xlsx

The ‘InterventionData’ dataset has four primary rows: ‘Participant’s identification number,’ ‘Condition,’ ‘Intervention Type,’ and ‘Timestamp of Intervention.’ Within the ‘Intervention Type,’ are three categories: ‘Discrete,’ ‘No Need for Intervention,’ and ‘Continuous Intervention for All Time.’ The ‘Discrete’ category captures interventions that occur at specific moments, ‘No need for Intervention’ indicates that intervention is not required during the entire SG, and lastly ‘Continuous Intervention for All Time’ denotes that interventions were consistently required during the entire SG.

#### Session Elapsed Time.xlsx

The ‘Session Elapsed Time’ represents the time spent in each session of the SG, measured in seconds. The data files include ‘Condition,’ ‘Participant’s identification number,’ and ‘Elapsed Time per Session.’

## Technical Validation

To validate our Engagnition dataset^53^, we implemented technical validations on the distributions of annotated engagement and intervention, and the SNR of physiological signals. First, we delineated the distribution of engagement annotation values for all study participants across two distinct physical exertion conditions (i.e., LPE and HPE), as outlined in Table 5. The distribution of annotated engagement values varies between study participants. While some participants consistently exhibited an engagement annotation coded 2, others showed a more diverse distribution across codes 0, 1, and 2. Overall, code 2 engagement was the most prevalent, followed by codes 1 and 0, in that order.

**Table 5:** Distributions of annotated engagement values (i.e., code 0,1, and 2) across LPE and HPE conditions: code 0 denotes participant is not engaged and out of control, code 1 indicates moderately engaged, and code 2 signifies highly engaged.

Condition	Annotation code (0)	Annotation code (1)	Annotation code (2)
LPE (%)	83,069 (28.38%)	57,899 (19.78%)	151,666 (51.82%)
HPE (%)	31,835 (7.17%)	161,814 (36.43%)	250,508 (56.4%)
Total (%)	114,904 (15.59%)	219,713 (29.82%)	402,174 (54.58%)

In addition, we categorized the number of interventions for each participant in a tabulated format (see Table 6). Out of the 38 participants in the LPE and HPE conditions, 6 required continuous interventions throughout the sessions. Excluding these 6 participants, the frequency of interventions between participants varied widely, ranging from a minimum of 0 to a maximum of 66 interventions. In-depth temporal analysis of each intervention are in the ‘InterventionData.xlsx’ file.

**Table 6:** The number of interventions required for each participants: The term “Continuous” signifies that participants engaged in the SG with assistance and intervention from the therapist during data collection.

LPE	Participant	P20	P21	P22	P23	P24	P25	P26	P27	P28	P29
	Number	0	0	30	0	2	0	Continuous	Continuous	33	Continuous
	Participant	P30	P31	P32	P33	P34	P35	P36	P37	P38	
	Number	5	0	0	0	0	40	15	0	23	
HPE	Participant	P39	p40	P41	P42	P43	P44	P45	P46	P47	P48
	Number	0	37	7	21	2	Continuous	4	1	2	Continuous
	Participant	P49	P50	P51	P52	P53	P54	P55	P56	P57	
	Number	0	0	0	0	66	0	Continuous	9	44	

Lastly, we assessed the quality of physiological signals (GSR and ST) from the Empatica E4 device using the SNR. To determine the SNR, we employed the autocorrelation function and used a second-order polynomial for a precise fit. Each set of physiological data was individually analyzed for its SNR using the decibel (dB) unit. For this estimation, we relied on the original, unaltered signals. Comprehensive statistics on SNR values are presented in Table 7. For GSR, the average SNR was 26.67 dB, with a variation of 3.48 dB. The median SNR for GSR was 27.03 dB, ranging from a low of 14.96 dB to a high of 34.29 dB. ST had an average SNR of 30.17 dB, with a variation of 3.20 dB. Its median SNR was 29.91 dB, and all values were between 13.28 dB and 36.02 dB. Additional subgroup details are available in Table 4. In line with our SNR analysis, the Empatica E4 data affirmed the high quality of the signals.

**Table 7:** Statistics on signal-to-noise ratio (SNR) of the physiological signal acquired via Empatica E4 wristband, Q means quantiles, All the SNR values are given in dB.

Condition	Sensor	Mean	Std	Min	Q15	Q25	Q50	Q75	Q95	Max
Baseline	GSR	26.74	2.80	20.18	23.73	24.52	27.23	28.95	30.32	30.75
	ST	29.41	0.71	28.06	28.80	29.02	29.43	29.77	30.26	31.38
LPE	GSR	26.71	3.10	19.33	24.50	25.23	26.74	28.07	30.58	34.29
	ST	29.43	2.30	25.89	27.27	27.53	28.61	31.11	33.48	33.50
HPE	GSR	26.57	4.34	14.96	23.37	24.49	27.03	30.27	31.98	32.04
	ST	31.68	4.63	13.28	30.67	31.32	32.49	33.71	35.25	36.02
Total	GSR	26.67	3.48	14.96	23.59	24.64	27.03	28.85	31.34	34.29
	ST	30.17	3.20	13.28	28.15	28.83	29.91	31.87	34.72	36.02

## Usage Notes

To optimize the processing of the Engagnition dataset^53^, we recommend the use of several Python libraries known for their effectiveness in preprocessing and feature extraction of physiological data. The Numpy library (https://numpy.org) plays a fundamental role in feature derivation from ACC, GSR, and ST signals. The SciPy library (https://scipy.org), equipped with a range of signal processing algorithms, including signal filtration, is essential for refining GSR signals. Additionally, the Pandas library (https://pandas.pydata.org) excels in resampling psychological signals at specific intervals. The Ledalab library focuses on GSR signals, providing both continuous and discrete decomposition analyses. These processing methodologies encompass engagement prediction, intervention time prediction, feature distillation, and the application of machine- and deep-learning algorithms.

The Engagnition dataset^53^ is exclusively reserved for academic research purposes, in adherence to the stipulations set forth by the data contributors. Individuals or entities desiring to use the dataset must first accede to the End User License Agreement (EULA) located within the repository of the dataset. Upon executing this agreement, the signed document is forwarded to the Engagnition Research Group via kimwon30@gm.gist.ac.kr, ensuring that correspondence is conducted through an official academic email address.

## Data Availability

The Engagnition software is available to the public through its official repository (https://github.com/dailyminiii/Engagnition). This repository mainly includes the code for analyzing data distribution and technical validation.
